# Impact of COVID-19 on Florida family dependency drug courts

**DOI:** 10.1186/s40352-024-00260-1

**Published:** 2024-02-08

**Authors:** Olivia K. Golan, Fatema Z. Ahmed, Barbara Andraka-Christou, Rachel Totaram, Yara Asi, Danielle Atkins

**Affiliations:** 1 NORC at the University of Chicago, Chicago, IL USA; 2 School of Global Health Management & Informatics, University of Central Florida, Orlando, FL USA; 3 Department of Internal Medicine, University of Central Florida, Orlando, FL USA; 4 Askew School of Public Administration and Policy, Florida State University, Tallahassee, FL USA; 5 Georgia State University, Atlanta, GA USA

**Keywords:** Family dependency drug courts, Problem-solving courts, Family treatment court, Drug court, COVID-19, Substance use disorder, Telehealth

## Abstract

**Background:**

To promote parent-child reunification, family dependency drug courts (FDDCs) facilitate substance use disorder treatment for people whose children have been removed due to parental substance use. The COVID-19 pandemic disrupted FDDC operations, forcing FDDCs to quickly adapt to new circumstances. Although existing research has examined COVID-19 impacts on adult drug courts and civil dependency courts, studies have yet to examine the impact of COVID-19 on FDDCs specifically.

**Methods:**

To explore the impact of COVID-19 on FDDCs, we conducted 20 focus groups and 5 individual interviews with court team members from five Florida FDDCs between 2020 and 2022. Data were analyzed using iterative categorization.

**Results:**

Five overarching themes emerged. First, FDDCs adopted virtual technology during the pandemic and more flexible drug screening policies. Second, virtual technology was perceived as improving hearing attendance but decreasing client engagement. FDDC team members discussed a potential hybrid in-person/virtual hearing model after the pandemic. Third, COVID-19 negatively impacted parent-child visitation opportunities, limiting development of bonds between parents and children, and parent-child bonding is a key consideration during judicial reunification decisions. Fourth, COVID-19 negatively impacted the mental health of court team members and clients. Court team members adopted new informal roles, such as providing technical support and emotional counseling to clients, in addition to regular responsibilities, resulting in feeling overwhelmed and overworked. Court team members described clients as feeling more depressed and anxious, in part due to limited visitation opportunities with children, which decreased clients’ motivation for substance use recovery. Fifth, COVID-19 decreased recruitment of potential clients into FDDCs.

**Conclusions:**

If FDDCs continue to rely on virtual hearings beyond the pandemic, they must develop practices for improving client engagement during virtual hearings. FDDCs should preemptively develop procedures for improving parent-child visitation during future public health crises, because limited visitation opportunities could weaken parent-child bonding and, ultimately, the likelihood of reunification.

## Introduction

At the beginning of the COVID-19 pandemic, courts nationwide were challenged by social distancing requirements and were required to modify court programs, shifting to virtual or contactless service delivery (Hartsell & Lane, 2022; Smith et al., 2022; Zilius et al., 2020). Problem-solving courts and dependency courts are two subsets of courts that faced similar service modifications during the pandemic. In contrast to traditional courts, which primarily handle civil and criminal disputes in an adversarial manner, problem-solving courts seek to address substance use or mental health issues as the root causes of criminal activity or child maltreatment. Problem-solving courts (sometimes called “treatment courts”) use a “hands-on” therapeutic jurisprudence approach with an interdisciplinary court team led by a judge to provide wrap-around services to court clients, including regular court hearings, treatment mandates, and monitoring, to address underlying causes of criminal/civil problems, including substance use disorder (SUD) (DeMatteo et al., 2019). Dependency courts (sometimes called “family courts”) address custody cases, including when parents have had children removed due to SUD issues (DeMatteo et al., 2019).

At the intersection of these two subsets of courts are family dependency drug courts (FDDCs; sometimes called “family treatment courts”), which use a therapeutic jurisprudence approach to address SUD as an underlying cause of child removal. FDDCs are specialized court programs targeting families impacted by SUDs within the child welfare system. These courts seek to enhance child well-being by delivering comprehensive services to parents with SUDs, with the goal of facilitating family reunification. Employing an integrated approach, FDDCs aim to disrupt the cycle of substance use and child maltreatment by integrating SUD treatment, parenting education, and ancillary support services into the legal framework. Operated collaboratively, FDDCs involve judges, attorneys, child welfare professionals, and SUD treatment providers. Court clients undergo comprehensive assessments to identify their specific needs, and individualized treatment plans are developed. FDDCs use a phased approach with duration contingent on factors such as SUD severity, parental treatment progress, and compliance with court directives. As compared to traditional dependency courts, clients in FDDCs are more likely to be reunified with their children (Zhang et al., 2019).

Literature on the effects of COVID-19 on problem-solving courts overwhelmingly focuses on criminal problem-solving courts, such as adult drug courts, instead of FDDCs. That literature suggests that virtual court service adoption during the pandemic had some positive outcomes, including reduced transportation barriers for court clients and improved convenience of virtual court team meetings (Hartsell & Lane, 2022; Zilius et al., 2020). The literature, however, also notes some challenges associated with virtual services, including technology barriers (Baldwin et al., 2020; Zilius et al., 2020), decreases in the number of clients referred to drug courts from other government institutions (Zilius et al., 2020), lapses in drug testing (Hartsell & Lane, 2022; Smith et al., 2022; Zilius et al., 2020), difficulty monitoring clients virtually (Hartsell & Lane, 2022), challenges intervening on a timely basis to prevent a return to drug use (Hartsell & Lane, 2022), disengagement in virtual SUD treatment (Hartsell & Lane, 2022), and distractions during virtual court sessions (Zilius et al., 2020).

Literature on dependency courts has likewise examined the effects of the pandemic and changes to virtual services, but it is focused on traditional dependency courts rather than FDDCs. Some states banned in-person visitation during the pandemic, and studies have found that traditional dependency courts suspended nearly all in-person visitation (Goldberg et al., 2021; Pisani-Jacques, 2020), potentially disrupting parent-child bonds and delaying reunification (Goldberg et al., 2021). In general, studies found that virtual visitation was not considered a suitable substitute for in-person visitation, especially after considering the young age of some children, developmental needs, technology utilization problems, and disparate access to technology (Goldberg et al., 2021; Oehme et al., 2021; Pisani‐Jacques, 2020). In one such study, court clients especially lamented the lack of physical contact with their children, and court team members reported that it was more difficult to gauge parenting skills and competency virtually (Oehme et al., 2021). Court clients also encountered pandemic-related challenges to employment, financial security, and education along with fears of older caregivers contracting COVID-19 (Font & Bartholet, 2021). Nevertheless, the study reported that virtual approaches to parent-child visitation increased flexibility and reduced transportation and scheduling difficulties (Oehme et al., 2021), ultimately reducing the total number of visitation “no-shows” and cancellations, resulting in a higher quantity of visits overall (Oehme et al., 2021).

While the literature has examined the effect of COVID-19 on criminal problem-solving courts (e.g., adult drug courts) and non-problem-solving courts (e.g., dependency courts), we are unaware of any work examining the effect of the pandemic at the intersection of these two types of courts – FDDCs. Therefore, the aim of this study was to examine the pandemic’s effects on the unique setting of FDDCs using focus groups and individual interviews with court team members in five Florida FDDCs between 2020 and 2022.

## Methods

We used the Consolidated Criteria for Reporting Qualitative Research (Tong et al., 2007) to report our research.

### Ethics

This research was approved by the Institutional Review Board at [university name deleted for blind review]. Study participants gave verbal consent prior to participation.

### Court sample

This study was part of a larger project evaluating the implementation of evidence-based parenting, mental health disorder (MHD), and SUD interventions in five Florida FDDCs. As a condition of receiving grant funding to implement evidence-based interventions, each of the five FDDCs were required to participate in evaluation activities with our research team.

### Instrument development

For the larger project, our research team developed a semi-structured focus group instrument with a series of questions about FDDC team members’ experience with implementing grant-funded interventions (e.g., parenting classes, peer support specialists) in their FDDC. A few months into the project, the onset of the COVID-19 pandemic led to a subset of questions being added to the focus group instrument to explore the impact of the pandemic on FDDC services, FDDC team members, and FDDC clients. For example, two of the questions added to the instrument were, “How has the COVID-19 pandemic impacted court operations?” and “What effects has the COVID-19 pandemic had on participants in your court?”

### Data collection

The PI recruited FDDC team members from each of the five Florida FDDCs receiving grant-funded interventions for focus groups. The PI emailed each FDDC’s coordinator, and the Florida Office of the State Courts Administrator (OSCA) sent reminder recruitment emails on the research team’s behalf. The recruitment email recommended that any FDDC court team members involved in the implementation of the grant-funded interventions participate in data collection.

Data were collected four times from each of the five FDDCs over three years, from 2020 to 2022. Each focus group session was limited to team members of one FDDC. In total, we conducted 20 focus groups, each with between two and 11 FDDC team members, as well as five individual interviews when it was not possible to convene an entire FDDC team due to schedule conflicts.

Recruitment shifted from in-person to virtual due to the COVID-19 pandemic, with three focus groups conducted in-person in conference rooms of the respective courts, and the remaining focus groups and interviews held virtually via Zoom software. Focus groups and interviews were conducted by qualitatively-trained masters’ or doctorate level researchers. Focus groups and interviews were audio-recorded with permission, and audio recordings were professionally transcribed. In compliance with governmental policy, FDDC team members were not provided a financial research incentive.

### Data analysis

Focus group and interview data were analyzed using iterative categorization (Neale, 2016) as follows. The research team created an *a priori* codebook based on a preliminary review of transcripts and the research questions. The codebook was then refined and entered into Dedoose qualitative software. Two research team members then independently coded meaningful excerpts of data and met to discuss differences in coding application, arriving at a final coded version. The codebook was iteratively adjusted during coding to reflect themes inductively emerging from the data. The final codebook included the parent code “COVID effect on court” with five sub-codes: “low engagement/recruitment,” “delays/interruptions of interventions,” “changing policy/practices at facility,” “parental visitation altered,” and “staff/client stressors.”

Data from each code were then exported into Excel worksheets (one worksheet per code), with a separate row for each excerpt. One qualitatively trained PhD-level researcher then labeled each excerpt with a summary of key points. For example, one paragraph of data might be summarized as “Virtual visitation is lower quality than in-person visitation because children have short attention spans.” That researcher then examined the summaries for consistencies and inconsistencies, thereby identifying patterns in the coded text, resulting in a code-level summary of key points. Finally, the researcher and project PI reviewed overarching themes across the code summaries, and other team members provided feedback on the interpretation of the results.

## Results

The five FDDCs were located in different regions of Florida, with three in urban areas and two in rural areas. Across the five FDDCs, a total of 72 court team members participated in at least one focus group or interview, including 6 from Court A, 24 from Court B, 10 from Court C, 14 from Court D, and 18 from Court E. In total, the following court team members participated in focus groups and/or interviews during the multi-year time period: case managers (*n* = 15), guardians ad litem (GAL) and non-GAL child advocates (*n* = 9), client advocates (*n* = 8), Department of Children and Families attorneys (*n* = 4), court coordinators (*n* = 11), SUD treatment providers (*n* = 11), and judges (*n* = 6). Eight court team members did not indicate their role (*n* = 8). All court team members who attended the focus groups contributed to the discussions to at least some extent, but judges and court coordinators tended to say the most.

Five overarching themes emerged. First, FDDCs adopted virtual technology during the pandemic and more flexible drug screening policies. Second, virtual technology was perceived as improving hearing attendance but decreasing client engagement. FDDC team members discussed a potential hybrid in-person/virtual hearing model after the pandemic. Third, COVID-19 negatively impacted parent-child visitation opportunities, limiting development of bonds between parents and children – a key consideration during judicial reunification decisions. Fourth, COVID-19 negatively impacted the mental health of court team members and clients. Court team members adopted new informal roles, such as providing technical support and emotional counseling of clients in addition to their regular responsibilities, resulting in feeling overwhelmed and overworked. Court team members described clients as feeling more depressed and anxious, in part due to limited visitation opportunities with children, which decreased clients’ motivation for substance use recovery. Fifth, COVID-19 decreased recruitment of potential clients into FDDCs.

### Theme 1: FDDCs adopted virtual services and became more flexible with drug screening

Some pandemic-related policies affecting court clients and court team members were imposed by the state or county rather than by the court, including stay-at-home orders and prohibitions on in-person parent-child visitation. The primary pandemic-related policies under the control of the court that emerged in our data related to the use of virtual technology and changes in drug screening practices.

All courts in our study adopted virtual hearings and virtual staffings. All courts also permitted virtual treatment. However, courts differed somewhat regarding their technology policies, such as whether audio-visual technology was required during court hearings. Ultimately, each court adopted one of the following policies for virtual court hearings: (1) court clients must use audio-visual technology (e.g., Zoom) unless they lack the technical capability to do so (e.g., they have no camera), in which case they can use audio-only technology (e.g., telephone); or (2) court clients may use either audio-visual or audio-only technology, depending on their preference and comfort. For example, a team member in Court D said:

*“It’s Zoom with video. Once in a while we’ll have somebody that can’t get their technology figured out but they should be on Zoom with video.” –* Court Coordinator, Court D

The former approach was primarily driven by the desire to ensure client engagement during the hearing. The latter approach was primarily driven by clients lacking computers and only possessing mobile phones – on which audio-visual calls and troubleshooting are difficult. For example, a team member in Court A explained:

*“I think a lot of our clients have smart phones, but it’s kind of a weird thing to hold and have all these tiles. If a phone is the device that you connect with, I think that people just generally join by phone… It’s a lot easier for us to troubleshoot with a phone. It’s a lot easier for us to say, ‘Yeah. Never mind. There’s a phone number with a participant code.’ We’re not IT people.”* – Court Coordinator, Court A

In addition to policies regarding virtual services, courts also had control over the extent to which they mandated or used urine drug screening (UDS) – an activity that was significantly impacted by COVID-19. Rather than requiring court clients to come to court for random UDS multiple times per week, during the pandemic courts resorted to temporarily stopping or reducing the frequency of UDS, requiring testing to be completed at alternate facilities, or relied on client self-reports of drug use only. Court team members noted that self-reports of drug use were unreliable.

*“Of course we couldn’t enforce them to go and drug test… So unfortunately, we weren’t able to get them tested. We had to rely on their self-report… One of our clients did say she didn’t use, but the next day she was transported to the hospital for an overdose. So self-reporting doesn’t always work.”* – Court Coordinator, Court A

### Theme 2: Virtual technology improved hearing attendance but decreased client engagement

Virtual provision of FDDC services was generally perceived as improving FDDC client access to hearings and treatment. FDDC team members frequently noted that virtual services alleviated the need for transportation, additional childcare, or taking time off from work, which led to increased attendance and participation in FDDC services. Alleviation of the need for transportation was particularly beneficial to FDDC clients in rural counties. For example, one FDDC team member described the convenience of virtual hearings for FDDC clients as follows:

*“These families are challenged and their resources are small and it’s hard to get places and they have a lot to do. Trying to get a job, see their kids, go to counseling, go to drug testing, work whatever job they’re working. This is great. We have one participant that just takes a 15-minute break and that’s how she gets on her hearing. That helps her instead of taking the whole day off.”* – Court Coordinator, Court D

FDDC team members perceived that clients greatly appreciated the conveniences of virtual FDDC services. One FDDC team member explained:

*“The only thing that [FDDC clients] value a lot of the times is their own time when it comes to coming to court and participating in treatment. It’s all about how much time, how much of their precious and personal time we’re all sucking. Whether it’s six hours of outpatient treatment a week, plus the two hours of individuals, plus the pro-social activities, plus you’ve got to come to court, all this, all this. We’ll suck 24 hours out of your life, but if we can get rid of four hours, because you don’t have to come to court, travel there, and appear, they really appreciate that as an incentive, like their time; Getting to see their time back.”* – Court Coordinator, Court A

The fact that virtual services led to more frequent hearing attendance made FDDC team members more aware of pre-existing barriers to in-person services. One FDDC team member summarized how their views of FDDC barriers have changed:

*“We’ve tried to get really creative. How do we overcome some of these barriers that are not necessarily the parents’ fault? And so, I think it’s made the system look at, as a whole, what we’re doing to try to think a lot more creatively and break down some of those barriers.”* – Case Manager, Court D

FDDC team members stated that court clients typically grasped how to use virtual technology easily. However, on some occasions, court clients faced barriers to using virtual technology for hearings, including lack of Internet access, limited computer access, and limited video and/or audio capabilities. Also, when court clients used mobile phones instead of computers, they sometimes had difficulty navigating video settings and other features, putting court team members in the informal role of “tech support.”

Despite improved access to FDDC services, FDDC team members felt that virtual technology generally worsened the quality of FDDC services. FDDC team members explained that it is more difficult to form personal connections with court clients virtually as compared to in person. Also, lack of in-person trips to the court house prevent informal interactions between court clients and FDDC team members. For example, court clients no longer crossed paths with FDDC team members organically when dropping off paperwork at the courthouse or during in-person drug screens and treatment appointments – times that FDDC team members describe as crucial informal opportunities for developing positive rapport with court clients. One FDDC team member described this challenge:

*“[Prior to the COVID-19 pandemic,] if somebody came in for a drug screen, you also chatted to them or if they came to bring paperwork in, you got to see them, so it seems like the staff now have to make a diligent effort to reach out and keep connecting, because they aren’t naturally just sort of coming in your path or interacting with you.”* – Case Manager, Court E

FDDC team members overwhelmingly noted a lack of engagement among court clients participating in hearings virtually (i.e., when clients appear before the FDDC judge and discuss their progress). For example, FDDC team members described observing court clients tapping between screens on their mobile phones or alternating between Zoom and other webpages while attending hearings. FDDC clients were also described as participating in virtual court hearings from locations that limit privacy or ability to focus, such as while in a car or walking in a public area. Relatedly, FDDC team members felt court clients were more likely to behave inappropriately or unprofessionally during virtual hearings, such as by smoking, lying in bed, or wearing casual clothing (e.g., pajamas). One FDDC team member said:

*“It would be nice to have, before they get involved with their services, for them not to appear from their bed, under their blankets, in a car as it’s moving… If they’re appearing outside that they’re not walking around so we’re staring at the trees and the skies swirling around and making us all dizzy. That’s really, for me as the court, the downside to remote hearings … there is a lack of formality.”* – Judge, Court B

Furthermore, FDDC team members felt court clients were less likely to pay attention to – and thus learn from – each others’ cases during virtual hearings. One FDDC team member said:

*“When you’re on virtual… it’s hard to gauge that as far as, ‘Are they really paying attention [to other clients], or are they just kind of waiting for their name called on the phone?’’* – Judge, Court A

Nevertheless, FDDC team members felt some clients were more comfortable participating in virtual treatment services compared to in-person services. For example, when talking about one court client, a counselor said:

*“Her fearfulness, her resistance to all of this is she is not open to talking to people…Our groups right now are all online and she was so comfortable… She was like, ‘Oh my God, this is not as bad as I thought it was going to be.’”* – SUD Treatment Provider, Court C

Some FDDC team members identified a desire to switch to a hybrid model in the future – one that incorporates both in-person and virtual services. FDDC team members anticipated that most court hearings would remain virtual, at least to some extent, post-pandemic. For example, one FDDC team member stated:

*“I do think that many aspects of what we envision traditional work will change post-COVID, whenever that happens. I believe that there will be a lot more virtual hearings especially the shorter ones, like the trials.”* – Judge, Court D

Some FDDC team members described a potential future hybrid “needs-based” model, wherein FDDC clients who have difficulty attending FDDC services in-person could use virtual technologies to bridge the gap and address existing barriers. For example, during our final year of data collection, one FDDC team member said:

*“Even though most [FDDCs] have gone back to in-person sessions, they have been able [to hold virtual sessions] now because the systems are set in place, ‘Oh, you live very far away or you’re having transportation problems. Okay, we will continue to do you through telehealth’ or something. So, I do think that those are going to be modifications that will continue to last.”* – Judge, Court E

FDDC teams differed about whether they would continue internal team meetings virtually after the end of the pandemic. Some felt virtual meetings were more efficient but that in-person meetings resulted in more effective communication between court team members.

### Theme 3: COVID-19 negatively impacted parent-child visitation and reunification in FDDCs

FDDC team members believed that COVID-19 negatively impacted parent-child visitation and reunification in FDDCs. Due to stay-at-home orders and/or restrictions on visitation imposed by the state or county, parents lacked opportunities to meet in person with their children. Even when such policies were lifted, FDDC team members said temporary caregivers (e.g., foster parents) were often unwilling to let children leave their residence. As a result, virtual parent-child visitation became necessary.

FDDC team members consistently described virtual visitation as being of lower quality than in-person visitation, negatively impacting the parent-child relationship and bonding opportunities. FDDC team members noted that judges consider the extent of parent-child bonding as one of several factors during final reunification decisions. For example, when asked about the extent to which parent-child bonding affects reunification decisions, a court team member said:

*“That’s usually probably one of the biggest things that weighs in on [reunification decisions], I would say… We look at the child’s, how comfortable they are with the parent when those visits are happening”* – Court Coordinator, Court C

For parents of young children, virtual visits were described as being especially problematic as compared to in-person visits. For example, parents could not hold or touch their infants during virtual visits – an age group that cannot interact virtually – and even older children frequently became distracted during video calls due to short attention spans. One court team member shared the story of an FDDC client who had been in residential SUD treatment for several months:

*“She has not had any in-person visits with her child the whole time she’s been [in residential treatment] because they are not allowing their transporters to transport children… So, mom has only had video contact with the child… her daughter is less than a year old… it’s super hard to communicate with an infant on video link. So that’s been super difficult… And she’s really, really doing well, but we can’t even talk about reunification because this child has no bond with her.”* – Court Coordinator, Court C

Relatedly, limited visitation was perceived by FDDC team members as harming SUD recovery, because clients’ desire to improve relationships with their children are often key motivators for treatment and abstaining from substance use. For example, one court team member explained:

*“When in-person visitation was suspended for a period of time, that made a huge impact on parents and their ability to bond and connect with their kids. And unfortunately, they’re not bonding and connecting, and … it doesn’t help with their sobriety. So, to that extent, I think that the pandemic really had an effect on how cases progressed or didn’t progress.”* – Court Coordinator, Court B

COVID-19 also caused some FDDC team members to reconsider traditional aspects of the reunification process/system. For example, the pandemic drew court team members’ attention to the multitude of barriers (e.g., transportation, childcare, employment navigation) that court clients must routinely overcome to meet FDDC requirements, as well as the large number of activities in which court clients must engage in aside from employment and visitation (e.g., attendance at court hearings, parenting classes, SUD treatment).

### Theme 4: COVID-19 had a negative impact on the mental health of FDDC team members and clients

FDDC team members believed that clients had become more depressed, anxious, stressed, hopeless, and overwhelmed during the pandemic, in part because they could not adequately connect and bond with their children via in-person visitation. One FDDC team member said:

*“I think it was just harder for those parents in residential because they were stuck, so many times they were under quarantine. When they were allowing them to go out somebody would come back with COVID… I think it kind of made them depressed or it made them give up… For them it was just like, ‘what am I doing? I’m not being rewarded. I still don’t have my kids.’”* – Case Manager, Court B

FDDC team members believed clients felt overwhelmed with FDDC program tasks combined with new challenges presented by the pandemic, including homeschooling any children who had not been removed from the home, finding/maintaining employment, adjusting to virtual services, finding transportation when public transportation shut down (e.g., to attend drug screenings), and managing COVID-19 illness in themselves and family members. One FDDC team member explained the following:

*“If you’ve got your kids getting COVID, or you’re getting COVID, and you’re having to not go to work, or you’re having to miss treatment, or you can’t show up for your drug screen, I think it definitely puts that extra barrier and challenge where they already have a lot anyway.”* – Court Coordinator, Court E

FDDC team members also said they themselves felt more overwhelmed, tired, and overworked during the COVID-19 pandemic. While working remotely, FDDC team members felt pressured to respond to work matters immediately and outside of standard work hours. During a focus group, two FDDC team members discussed:

*“Everybody’s working a lot more. You’re working from home, but I mean, you’re immediately responding. Now it’s expected for you to respond immediately. I used to drive 10 to 13 hours a week. Now, I’m doing work those 10 to 13 [hours]. It’s horrible. I can’t keep up with my Audible. Now, I’m behind on my books. So, that 10 hours to 13 hours is all work.”* – Case Manager, Court E

*“We have luckily a group of people who have a very strong work ethic, but also unluckily we have a group of people that have a very strong work ethic, so people are overworking because of it.*” – Court Coordinator, Court E

FDDC team members also expressed difficulty adjusting to constant program changes while simultaneously helping clients adjust to those program changes too, including by providing “tech support” to clients during hearings (e.g., when clients did not know how to use Zoom). In addition, FDDC team members described adopting a counselor-type role in addition to their regular duties during the pandemic, despite lacking training as a counselor, helping FDDC clients manage anxiety related to uncertainty and changing program expectations. For example, one FDDC team member described:

*“I think also my [clients] have anxiety to the roof. So, a lot of things that they have been used to, we had to navigate them and slowly tell them, okay, listen, A and B isn’t going to be A and B. We’re going to go to A and C, but it’s still expected of you and I to do the same things that we kept on doing. So that was a bit hard for us. And then we ended up being more of a therapist per se, which also did add on the extra work that we had to do”* – Case Manager, Court B

### Theme 5: COVID-19 negatively impacted FDDC recruitment of new FDDC clients

FDDCs are optional alternatives to traditional dependency courts. Most FDDCs in the study experienced significant declines in the number of clients recruited from traditional dependency courts into FDDCs after the pandemic began. Before the pandemic, FDDC team members would meet with and give presentations about benefits of FDDCs to child welfare agencies; but such meetings declined after child welfare agencies stopped in-person meetings, and courts were slow to adopt virtual meetings with child welfare agencies instead. For example, one FDDC team member stated:

*“We reach out to the case managers or their supervisor and ask them to kind of look at their caseload to determine whether or not there is some cases that they can refer over to us. Due to COVID, we have not been out. Prior to COVID, I would actually go to some of the meetings with our service providers and do presentations. But I haven’t done that yet. I haven’t been out and about. I probably should reach out and maybe do something over Zoom or something like that.”* – Court Coordinator, Court A

Prior to the pandemic, another common recruitment strategy for FDDCs occurred during shelter hearings – wherein FDDC team members sought to convince parents attending a traditional dependency court to opt into the voluntary FDDC instead. FDDC team members would, while in-person, describe the additional benefits of FDDCs, such as funded services for which parents would be eligible and a greater likelihood of reunification. Such conversations sometimes occurred immediately before or after shelter hearings. During the pandemic, FDDC team members tried recruiting clients via virtual shelter hearings instead, but FDDCs team members felt fewer opportunities existed during which to speak with potential FDDC clients and that virtual conversations were less persuasive than in-person conversations. One FDDC team member explained:

*“Over the last few years, there’s been a decline [in client recruitment] for a variety of reasons and factors, but I think really COVID is the biggest one for the last year. Normally we talk with participants every day when they come to their shelter hearing. We can tell them about the family drug court, providing information, and right now people are all virtual. So, even though you sometimes try to reach out to them over the phone … it’s not often that we connect with them so that recruiting component I think has been challenged by COVID.”* – Court Coordinator, Court D

## Discussion

Our multi-year study examined the effects of the COVID-19 pandemic on the practices of five FDDCs, as well as effects on court team members and court clients. We found that court team members were forced to quickly navigate the risks of a contagious virus and externally imposed stay-at-home orders through adoption of virtual court hearings and flexible drug screening models. Unfortunately, we also found that FDDC team members experienced increased mental health stressors, as did court clients, and that parent-child bonding was severely hindered by state-imposed limitations on in-person parent-child visitation.

During data collection, FDDC team members repeatedly discussed their experiences with virtual hearings, suggesting that the shift from in-person to virtual hearings was among the most salient and important aspects of courts’ pandemic experience. Generally, court team members in our study appreciated the convenience of virtual hearings, which led to more frequent client attendance at hearings. Another study also found that attendance at criminal hearings significantly increased after transitioning from in-person to virtual hearings, although access decreased for a small portion of participants who experienced difficulty obtaining Wi-Fi and/or necessary technology (Kunkel & Bryant, 2022).

Despite increased client attendance, court team members in our study expressed concerns about the quality of client engagement during virtual hearings. Low client engagement during hearings could translate into worsened relationships between clients and court team members; but such relationships are considered a critical piece of why problem-solving courts work (Clark, 2001; Dakof et al., 2010; National Association of Drug Court Professionals, 1997). If FDDCs transition to a hybrid service delivery model, then FDDC team members should develop ways to nurture relationships with clients virtually (e.g., offering virtual office hours). During our study, FDDC team members also noted that court clients attending virtual hearings were more likely to appear distracted, call from public areas (potentially limiting confidentiality), or behave unprofessionally. In the future, FDDCs could consider providing brief training to clients about professional/appropriate courtroom behavior during video conferencing – including the type of clothing to wear (e.g., not pajamas), use of virtual backgrounds, and distracting movement during videos on mobile phones. Therefore, if virtual FDDC hearings continue beyond the COVID-19 pandemic, FDDCs will need to find a balance between accessibility and quality of virtual court hearings. Some FDDC team members believed that hybrid court hearings are likely to occur in the future, although how such models will be operationalized remains to be seen. For example, FDDCs could implement a need-based hybrid model, wherein only FDDC clients who face transportation-related or other barriers are allowed to attend virtual hearings; or, FDDCs may opt for a merit-based model, wherein court clients are given virtual hearing privileges as they successfully advance through FDDC program phases. Future research should consider the ethics and feasibility of different hybrid models.

The primary purpose of FDDCs is to facilitate parent-child reunification through addressing underlying substance use and mental health issues among parents. We are troubled by the finding that the COVID-19 pandemic may have decreased the likelihood of parent-child reunification for FDDC clients, specifically by limiting in-person parent-child visitation, through which parent-child bonding occurs. In fact, the existence of parent-child bonding is among the key factors considered by judges making reunification decisions (Center for Children and Family Futures and National Association of Drug Court Professionals, 2019). Unfortunately, the state of Florida issued a moratorium banning in-person visitation for approximately eight months during the pandemic (Steering Committee on Families and Children in the Court, 2020).

FDDC team members create parent-child visitation plans with a frequency of visits sufficient to establish, maintain, and strengthen the parent-child relationship while ensuring the child’s safety. FDDC best practice standards specifically recommend “face-to-face” visitation (Center for Children and Family Futures and National Association of Drug Court Professionals, 2019). FDDC team members in our study emphasized the importance of in-person visitation for parents with newborns in particular, as newborns have no ability to bond virtually. Decreased parent-child visitation opportunities were even described as triggering return to drug use among some parents, who (at least temporarily) lost their main motivation for staying in recovery. Court team members in our study noted the near impossibility of newborns bonding with parents – and vice versa – during virtual visits.

Ample literature suggests the entire child welfare system faced similar difficulties as the FDDCs in our study, including weakened parent-child bonds and delayed reunification due to the cessation of in-person visitation (Goldberg et al., 2021; Pisani-Jacques, 2020). Unfortunately, it is likely that these short-term effects will lead to long-term harms, such as attachment issues (Pisani‐Jacques, 2020). Virtual visitations with incarcerated parents during the COVID-19 pandemic were similarly described as inferior to in-person visits, having negative impacts on the well-being of both children and their incarcerated parents (Flynn et al., 2022).

Relatedly, studies of parents with children in neonatal intensive care units (NICUs) during the pandemic reported significant negative impacts on parental well-being and parent-child bonding (Erdei & Liu, 2020; McCulloch et al., 2022). Notably, 90% of children’s brain development occurs before the age of three (Perry, 2000), and child-parent interactions during this time period play a crucial role in brain development, determining whether children will be able to form healthy attachments and regulate their own emotions later in life (Winston & Chicot, 2016). Future research should examine the downstream effects of prohibiting in-person visitation on parent and child well-being and child development during the COVID-19 pandemic. Researchers should also explore ways to mitigate social, emotional, and cognitive issues developed by FDDC clients and their children during the COVID-19 pandemic and prevent these issues during future public health crises, such as by creating opportunities for safe in-person visitation.

FDDC team member roles seemed to shift and expand during the pandemic. Some FDDC team members worked more, as remote work boundaries had yet to be established. Employee burnout was reported across nearly all professions and industries during the COVID-19 pandemic (Gabriel & Aguinis, 2022). FDDC team members also described adopting tech support and emotional support roles during the pandemic, and these shifting and expanding responsibilities further exhaustion in the FDDC workforce.

Recruitment was also an issue that arose for FDDCs. Courts had significant declines in recruitment of new clients during the pandemic compared to pre-COVID, primarily because FDDC team members decreased interactions with referring agencies and were unable to explain benefits of FDDCs in person to potential clients during shelter hearings. Going forward, courts should consider other recruitment methods besides in-person networking to educate referring agencies and potential clients on the role of FDDCs.

Limitations include a potential lack of generalizability outside of our sample, which was limited to five FDDCs that received grant funding in Florida. Another limitation is that our sample only included FDDC team members, not FDDC clients themselves, and FDDC clients could have different perspectives on the impact of COVID-19 on FDDCs. Future research should explore FDDC clients’ experiences in FDDCs during COVID-19. As compared to other court team members, court coordinators and judges spoke most frequently during our focus groups, likely reflecting the fact that they are rich information sources about court practices and policies; however, power dynamics wherein other team members implicitly defer to court leaders may also play a role and should be considered when interpreting results. Also, we did not collect information about FDDC team members who opted not to participate in this study, and therefore, we cannot determine if there was a systematic reason for participation or lack thereof. In addition, two courts opted to have team members participate in interviews instead of a focus group due to scheduling conflicts (Court A during Phase 1 and Court C during Phase 2). It is possible that court team members who participated in interviews would have divulged different information if they had participated in focus groups instead, although we did not identify systematic differences in responses or roles between those who were interviewed versus those in focus groups during our analysis. Relatedly, while we did not identify explicit disagreements among court team members during individual focus groups, we cannot be 100% certain that speakers’ statements during focus groups reflect the beliefs of their entire court team.

Despite these limitations, to our knowledge, this was the first study to examine the impact of COVID-19 specifically in FDDCs, with prior work focusing on the impact of COVID-19 on traditional dependency courts or other problem-solving courts only. Additionally, we collected qualitative data longitudinally over a three-year period following the onset of the COVID-19 pandemic, capturing a broader time range and potentially more nuanced perspectives than shorter-term studies exploring the impact of COVID-19 on criminal justice, child welfare, and SUD treatment services.

## Conclusion

Court team members seemed to have difficulty keeping pace with and handling shifts in state and county-level policies during the COVID-19 pandemic. Due to the sudden and unpredictable nature of the public health crisis, courts were forced to make operational decisions in real-time with limited opportunities to weigh benefits and challenges of competing options. Our results suggest that courts would benefit from regularly (e.g., perhaps annually) reviewing policies and procedures for functioning during crises, whether caused by pandemics, natural disasters, or terrorism. For example, drawing on lessons learned during the COVID-19 pandemic, courts could preemptively plan how to facilitate parent-child bonding, drug screening, and monitoring of court clients when stay-at-home orders are in place.

## Data Availability

Raw group and individual interview data are not publicly available to protect participants’ privacy.
